# Epigenetic biomarkers of ageing are predictive of mortality risk in a longitudinal clinical cohort of individuals diagnosed with oropharyngeal cancer

**DOI:** 10.1186/s13148-021-01220-4

**Published:** 2022-01-03

**Authors:** Rhona A. Beynon, Suzanne M. Ingle, Ryan Langdon, Margaret May, Andy Ness, Richard M. Martin, Matthew Suderman, Kate Ingarfield, Riccardo E. Marioni, Daniel L. McCartney, Tim Waterboer, Michael Pawlita, Caroline Relton, George Davey Smith, Rebecca C. Richmond

**Affiliations:** 1 MRC Integrative Epidemiology Unit, University of Bristol, Oakfield House, Bristol, BS8 2BN UK; 2 Population Health Sciences, Bristol Medical School, University of Bristol, Bristol, UK; 3 NIHR Bristol Biomedical Research Centre, University Hospitals Bristol and University of Bristol, Bristol, UK; 4 Centre for Trials Research, Neuadd Meirionnydd, Heath Park Way, Cardiff, UK; 5 Community Oral Health, University of Glasgow Dental School, Sauchiehall Street, Glasgow, UK; 6 Centre for Genomic and Experimental Medicine, Institute of Genetics and Molecular Medicine, University of Edinburgh, Edinburgh, EH4 2XU UK; 7 Infections and Cancer Epidemiology, German Cancer Research Center (DKFZ), Heidelberg, Germany

**Keywords:** Epigenetic clock, Epigenetic ageing, Oropharyngeal cancer, DNA methylation, Mortality, Prediction

## Abstract

**Background:**

Epigenetic clocks are biomarkers of ageing derived from DNA methylation levels at a subset of CpG sites. The difference between age predicted by these clocks and chronological age, termed “epigenetic age acceleration”, has been shown to predict age-related disease and mortality. We aimed to assess the prognostic value of epigenetic age acceleration and a DNA methylation-based mortality risk score with all-cause mortality in a prospective clinical cohort of individuals with head and neck cancer: Head and Neck 5000. We investigated two markers of intrinsic epigenetic age acceleration (*IEAAHorvath* and *IEAAHannum*), one marker of extrinsic epigenetic age acceleration (*EEAA*), one optimised to predict physiological dysregulation (*AgeAccelPheno*), one optimised to predict lifespan (*AgeAccelGrim*) and a DNA methylation-based predictor of mortality (*ZhangScore*). Cox regression models were first used to estimate adjusted hazard ratios (HR) and 95% confidence intervals (CI) for associations of epigenetic age acceleration with all-cause mortality in people with oropharyngeal cancer (*n* = 408; 105 deaths). The added prognostic value of epigenetic markers compared to a clinical model including age, sex, TNM stage and HPV status was then evaluated.

**Results:**

*IEAAHannum* and *AgeAccelGrim* were associated with mortality risk after adjustment for clinical and lifestyle factors (HRs per standard deviation [SD] increase in age acceleration = 1.30 [95% CI 1.07, 1.57; *p* = 0.007] and 1.40 [95% CI 1.06, 1.83; *p* = 0.016], respectively). There was weak evidence that the addition of *AgeAccelGrim* to the clinical model improved 3-year mortality prediction (area under the receiver operating characteristic curve: 0.80 vs. 0.77; *p* value for difference = 0.069).

**Conclusion:**

In the setting of a large, clinical cohort of individuals with head and neck cancer, our study demonstrates the potential of epigenetic markers of ageing to enhance survival prediction in people with oropharyngeal cancer, beyond established prognostic factors. Our findings have potential uses in both clinical and non-clinical contexts: to aid treatment planning and improve patient stratification.

**Supplementary Information:**

The online version contains supplementary material available at 10.1186/s13148-021-01220-4.

## Background

Oropharyngeal cancer (OPC), which includes cancers of the soft palate, base of tongue, uvula, palatine tonsils and tonsillar pillars [1], is the second most commonly diagnosed head and neck cancer (HNC) in the UK, with an age-standardised incidence rate of 2.9 per 100,000 persons [2]. Risk factors include smoking, alcohol consumption and human papillomavirus (HPV) infection. Estimated 5-year survival rates for people with OPC vary from 35 to 83% [3, 4]. As such, the ability to estimate survival probabilities at the time of diagnosis is important for clinical decision making and enrolment of low-risk individuals into treatment de-escalation trials [5].

HPV positivity, primarily HPV16, is a major determinant of OPC prognosis [6–8]. Compared to people with non-HPV-driven tumours, people with HPV-driven tumours have a 60% reduced risk of death 3-year post-diagnosis [8]. Consequently, HPV status is now included in prognostic models alongside TNM stage and comorbidity [8–11]. One such model has yielded a Harrell’s concordance statistic (C-statistic) of 0.68 (95% confidence interval [CI] 0.65, 0.71) in external validation, indicating good (but not excellent) prediction [12]. The potential for model improvement is currently being explored and the prognostic value of lifestyle factors has been evaluated [13–19]. The prognostic role of epigenetic biomarkers is less well studied.

Epigenetic biomarkers of ageing (“epigenetic clocks”), which are multivariate predictors of biological age based on DNA methylation (DNAm) levels at a subset of CpGs across the genome, are demonstrating promise in predicting age-related disease and mortality [20–22]. Most studies evaluating the prognostic utility of these epigenetic clocks have been conducted in general (healthy) populations, however [22–24]. There is a paucity of studies focusing on clinical populations. One study used a Cox model to estimate hazard ratios (HRs) for the association between epigenetic age acceleration (EAA), that is the difference between age predicted by the epigenetic clocks and chronological age, and risk of death following cancer diagnosis (*n* = 1726 deaths) [25]. After adjusting for socio-demographic and lifestyle variables, the authors found limited evidence (OR 1.04, 95% CI 1.00–1.09) of an association with EAA based on an epigenetic clock derived from methylation at 353 CpG sites (*EEAHorvath*) [26]. However, mortality risk was 28% higher (OR 1.28, 95% CI 1.11–1.47) for the highest versus lowest quartile of age acceleration based on an epigenetic clock derived from methylation at 71 CpG sites (*EEAHannum*) [27].

In this study, we investigated six epigenetic biomarkers in relation to survival in a cohort of individuals with OPC (*n* = 408). We examined associations between both “first generation” epigenetic clocks derived from DNAm levels at CpG sites found to be strongly associated with chronological age, and two more recently derived clocks: one optimised to predict physiological dysregulation and one optimised to predict lifespan. We also examined the association of a DNAm-based mortality risk score with survival.

In stage one of our analyses, we examined the associations of the six epigenetic biomarkers with survival using cox regression models, with and without adjustment for factors known to influence epigenetic ageing. In the second stage, we implement flexible parametric survival models to investigate the added prognostic value of epigenetic markers compared to a standard clinical model that included age, sex, TNM stage and HPV status.

## Methods

### Study population

The study population included a subset of individuals with OPC enrolled in the Head and Neck 5000 (H&N5000) study, a prospective, UK-based, clinical cohort study of people with HNC (*n* = 5518) [28, 29]. H&N5000 was approved by the National Research Ethics Committee (South West Frenchay Ethics Committee, 10/H0107/57) on 5th November 2010 and approved by the Research and Development departments of participating NHS Trusts.

Individuals were selected based on pre-treatment clinical coding of OPC and the availability of baseline questionnaire and clinical data-capture information. Where possible, pathology reports of individual cases were subsequently checked to verify tumour site and subtype. Overall, 5474/5518 (99%) data-capture forms were completed, and 3361/5385 (62%) individuals returned all three baseline questionnaires.

### Baseline data collection

Consent was wide-ranging, including permission to: collect, store and use biological samples; carry out genetic analyses; collect information from hospital records and through self-reported questionnaires; and obtain mortality data through electronic record linkage [28]. Baseline collection was completed pre-treatment, unless the individual’s diagnosis and treatment were the same procedure (e.g. tonsillectomy), in which case recruitment and baseline procedures were completed within a month of the diagnostic procedure. Blood samples (*n* = 4676, 85%) were sent to the study laboratory (https://www.bristol.ac.uk/population-health-sciences/research/groups/bblabs/) at ambient temperature for processing. They were centrifuged at 3500 rpm for 10 min and the buffy coat layer used for DNA extraction. Additional samples were frozen and stored at − 80 °C.

### Assessment of HPV status

HPV serologic testing for HPV16 (E6, E7, E1, E2, E4 and L1) antibodies was conducted at the German Cancer Research Center (DKFZ) using glutathione S‐transferase multiplex assays. HPV16 E6 seropositivity (a marker of HPV‐transformed tumour cells [30]) was indicated if HPV16 E6 median fluorescence intensity (MFI) was > 1000 units [31, 32].

### DNA methylation profiling

**Fig. 1:**
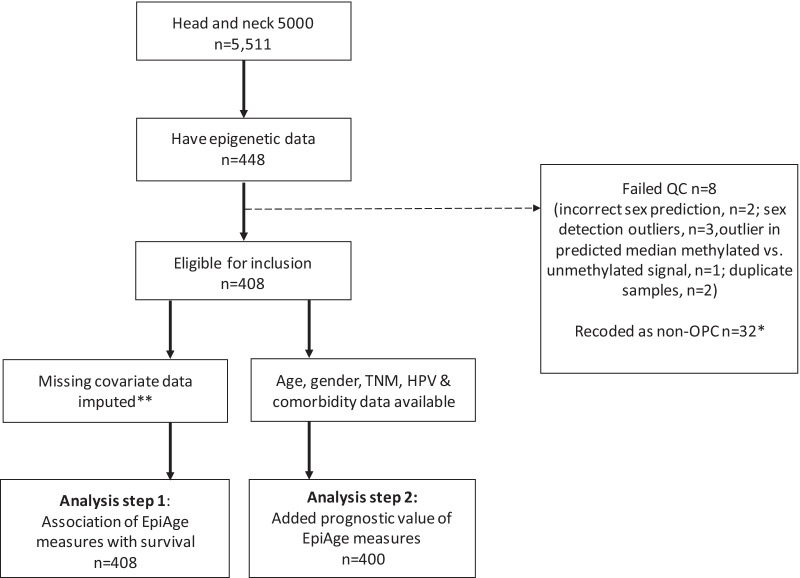
Flow of participants included in the analysis. OPC, oropharyngeal cancer; QC, quality control

DNA was bisulphite-converted using the Zymo EZ DNA Methylation™ kit (Zymo, Irvine, CA, USA) and genome-wide methylation data were generated using the Infinium MethylationEPIC BeadChip (EPIC array; Illumina, USA). Raw data files were pre-processed using the R package *meffil* (https://github.com/perishky/meffil/) [33]. Overall, 440/448 samples passed quality control and were normalised (Fig. 1). Further details are provided in the Supplementary Material (Additional file 3).

### Estimation of epigenetic age

DNAm data for a subset of CpGs on the EPIC array (*n* = 27,523) and an annotation file containing data on chronological age, sex and tissue type were uploaded onto the DNAm Age Calculator https://dnamage.genetics.ucla.edu/ (Additional file 3: Supplementary Methods). The following epigenetic ageing measures were obtained: intrinsic epigenetic age acceleration based on Horvath’s multi-tissue predictor (*IEAA*) [26]; intrinsic epigenetic age acceleration based on Hannum’s predictor (*IEAAHannum*) [27]; extrinsic epigenetic age acceleration (*EEAA*), an enhanced version based on Hannum’s method, which up-weights the contribution of blood cell composition [21]; PhenoAge (*AgeAccelPheno*) [34] and GrimAge (*AgeAccelGrim*) [35] An overview of the age predictors is provided in Table 1. In each case, age acceleration was defined as the residual obtained from regressing predicted age, as estimated by the epigenetic clock, on chronological age.

**Table 1:** Overview of various measures of epigenetic age acceleration and mortality risk used in this analysis

Epigenetic marker	Abbreviation	CpGs	Description	References
Intrinsic Epigenetic age acceleration based on Horvath	*IEAA*	353	The residual resulting from regressing DNAm age on chronological age and estimates of major blood immune cell counts ^*^	[26]
Intrinsic epigenetic age acceleration based on Hannum	*IEAAHannum*	71		[27]
Extrinsic age acceleration based on Hannum	*EEAA*	71	The residual resulting from a univariate model regressing a weighted age estimate (which increases the contribution of 3 cell types known to change with age ^**^) on chronological age	[21]
Age acceleration based on PhenoAge	*AgeAccelPheno*	513	The residual resulting from a linear model when regressing *PhenoAgeAccel* on chronological age, where PhenoAge is an ageing measure based on a linear combination of chronological age and nine clinical biomarkers	[34]
Age acceleration based on GrimAge	*AgeAccelGrim*	1030	The residual resulting from a linear model when regressing GrimAge on chronological age, where GrimAge is an ageing measure based on a linear combination of chronological age, sex and DNAm-based surrogate biomarkers for smoking pack-years (*DNAmpackyears)* and seven plasma protein levels	[35]
Mortality risk score based on Zhang	*ZhangScore*	8	A linear combination of LASSO regression coefficient weighted methylation values of the ten CpGs	[36]

^*^ Naive CD8 + T cells, exhausted CD8 + T cells, plasmablasts, CD4 + T cells, natural killer cells, monocytes and granulocytes. ^**^ naïve (CD45RA + CCR7 +) cytotoxic T cells, exhausted (CD28-CD45RA-) cytotoxic T cells and plasmablasts

### Generation of the DNAm-based mortality predictor in H&N5000

The epigenetic predictor for mortality (*ZhangScore*) was generated using the equation in [36]. Two of the ten CpGs included in the DNAm score were not present in the H&N5000 epigenetic data because methylation was measured using the EPIC array rather than Illumina450K array, on which the score was developed. The score was therefore generated using the remaining 8 CpGs (See Additional file 3: Supplementary Methods).

### Study follow-up and survival

Regular vital status updates were received from the NHS Central Register and NHS Digital, notifying on subsequent cancer registrations/deaths among cohort members. Recruitment finished December 2014 and follow-up information on survival status was obtained on 1 September 2018. The median duration of follow-up was 4.3 years (inter-quartile range [IQR] 3.3–5.2).

### Covariates

Information on age at diagnosis, sex, weight, height, marital status, highest educational attainment (school education, college or degree-level), annual household income, smoking status (defined as “current”, “former” or “never” user of tobacco) and alcohol intake (units per week) were obtained from baseline questionnaires, which are available on the study website (http://www.headandneck5000.org.uk/). We were unable to include lifetime exposure to tobacco (i.e. pack-years) in the current analysis because information relating to time since starting and time since quitting smoking was insufficient, i.e. not enough people completed these questions at baseline. Furthermore, the questionnaire did not capture information regarding periods of abstinence from tobacco use.

Clinically meaningful alcohol drinking categories (both sexes) were defined as “none”, “moderate” (≤ 14 units/week) and “hazardous-to-harmful” (> 14 units/week), based on UK guidelines [37] (Additional file 3: Supplementary Methods). We used categories of alcohol intake in our main analyses (rather than units consumed) because categories of drinking form the basis of clinical advice, i.e. they are more clinically relevant, and many governments and public health bodies have sought to promote public guidelines for “low risk” or “sensible” drinking based on cut-offs of intake. In addition, these alcohol exposure variables are consistent with previous publications [17, 38].

Body mass index (BMI) was calculated as: weight (kg)/(height (m))^2^. Comorbidity was defined as “none”, “mild”, “moderate” or “severe” based on the extent of functional deterioration, as measured by the ACE-27. Ethnicity was not included because only two individuals reported being non-white.

Sex, diagnosis, stage and comorbidity were recorded on the data-capture form. Diagnosis was coding using the International Classification of Diseases (ICD) version 10 [39]. Clinical staging of the tumour from T (characteristics of the tumour site), *N* (degree of lymph node involvement) and M (the absence or presence of metastases) were based on the American Head and Neck Society TNM staging of head and neck cancer [40]. Comorbidity was determined using the Adult Comorbidity Evaluation-27 [ACE-27] [41].

### Statistical analysis

Stata 15.0 (StataCorp. 2017) was used for all analyses. Firstly, we examined whether EAA measures were associated with survival, after controlling for established HNC prognostic factors; secondly, we investigated whether these measures provide any additional prognostic information, over and above factors that are considered in routine clinical practice.

#### Step 1: examining associations of EAA measures with survival

Descriptive analyses were performed to explore the distribution of, and correlations between EAA measures. Baseline descriptive data were stratified by survival at 3 years. The univariate association of covariates on all-cause mortality risk was assessed using Kaplan–Meier curves and log-rank tests.

Multivariable Cox proportional hazards models were used to examine associations of EAA measures and the mortality predictor with overall survival, defined as the time in years from study enrolment to date of death from any cause or date of censorship (i.e. the last date of follow-up). Measures were standardised using z-scores to allow comparison of effect estimates. Hazard ratios (HRs) and 95% CIs for all-cause mortality were calculated for each standard deviation (SD) increase in EAA.

For each epigenetic ageing marker, four separate Cox models were run: (1) a minimally adjusted model that controlled for sex; (2) a model that additionally controlled for clinical factors (TNM stage, HPV status, comorbidity and BMI); (3) a model that additionally controlled for socio-demographic and economic factors (education, annual household income, marital status) and (4) a fully adjusted model that additionally controlled for lifestyle behaviours (self-reported smoking and alcohol consumption). Models were selected a priori based on the existing literature linking these covariates with survival [15, 42–46]. As a sensitivity analysis, we used the continuous measure of alcohol intake (units /week) rather than categories of intake in model 4.

For the DNAm-based mortality predictor (*ZhangScore*), the same models were run, with the exception that the minimally adjusted model also included age at time of diagnosis, since chronological age was not factored in when generating this score.

The proportional hazards assumption was checked using statistical tests and graphical diagnostics based on the Schoenfeld residuals. Missing covariate values were imputed using the ICE package for multiple chained equations in Stata [47] (Additional file 3: Supplementary Methods). As a further sensitivity analysis, we created a complete case dataset and analysed as above [48].

We chose not to include chronological age as a covariate in the (EAA) primary survival models because, by definition, age acceleration residuals from a DNAm age predictor should be zero (i.e. not correlated with chronological age). However, since chronological age is positively correlated with mortality, we re-ran the cox models adjusting for chronological age (imputed and complete case).

#### Step 2: assessing the prognostic value of EAA measures

Evidence of an association with survival is not enough to include novel biomarkers in prediction models; to aid clinicians they must provide added prognostic value to existing models [49]. We explored whether the addition of EAA measures to existing models based on established mortality risk factors (i.e. those currently considered in clinical decision making), improved model performance.

Flexible parametric survival models were fitted using the methods of Royston and Parmar [50, 51] (Additional file 3: Supplementary Methods). Models were fitted using maximum likelihood estimation via the “stpm2” command. Nonlinear relationships with continuous predictors were considered using the multivariable fractional polynomial (MFP) algorithm [52] and implemented in Stata using the “mfp” command.

The following models were fit: (1) a “clinical model”, which comprised age, sex, TNM stage, HPV status and comorbidity; (2) clinical + *IEAA*; (3) clinical + *EEAA*; (4) clinical + *IEAAHannum*; (5) clinical + *AgeAccelGrim*; (6) clinical + *AgeAccelPheno*; (7) clinical + *ZhangScore.* Models were fit in a sub-sample of participants with data available for the clinical covariates included in the model (age, sex, tumour stage, comorbidity and HPV status).

The performance measures examined were the Akaike Information Criterion (AIC) and the C-statistic, an extension of the area under the receiver operating curve (AUC) to survival analysis [53, 54]. ROC curves and AUC functions were also calculated to characterise how well the models distinguished between people who were and were not alive at 3 years. Internal validation was performed using 500 bootstrap samples to adjust performance for optimism and calculate a shrinkage factor to be applied to model regression coefficients. Where there was evidence of model improvement with addition of the epigenetic markers, assessed based on the C-statistic, we also examined the complementary role of these markers in the prediction of mortality through inclusion in the same model.

## Results

In total, 408 out of 1896 participants with pathologically confirmed OPC had epigenetic data available (Fig. 1). There were 105 deaths during follow-up (median = 5.3 years, IQR 4.9–6.0). The proportion of missing data is presented in Additional file 1: Table S1.

### Baseline descriptives

Participants who were alive at 3 years had a mean age of 57.4 years at diagnosis (SD = 8.9) compared to 62.9 years (SD = 11.3) (Table 2). Overall, mean EAA measures were lower in people who were alive. The mortality risk score was also lower in those individuals who were alive at 3 years. See Additional file 1: Table S2 for complete case descriptives.

**Table 2:** Baseline characteristics of the study sample stratified by 3-year mortality status (*n* = 408)

Characteristic	Overall (*n* = 408)		Dead at 3 years (*n* = 77)		Alive at 3 years		*p *value
					(*n* = 331)		
	*N*	*%*	*N*	*%*	*N*	*%*	
*Sex*							
Male	317	77.70	60	77.90	257	77.60	
Female	91	22.30	17	22.10	74	22.40	0.958
*TNM stage group*							
I	17	4.20	1	1.30	16	4.80	
II	39	9.60	4	5.20	35	10.60	
III	58	14.20	14	18.20	44	13.30	
IV	294	72.10	58	75.30	236	71.30	0.175
*HPV status*							
Negative	122	29.90	45	58.40	77	23.30	
Positive	286	70.10	32	41.60	254	76.70	< 0.001
Comorbidity status*							
None	211	52.10	26	34.20	185	56.20	
Mild	119	29.40	27	35.50	92	28.00	
Moderate/severe	75	18.50	23	30.30	52	15.80	0.001
*Smoking*							
Never	110	28.10	8	11.00	102	32.00	
Former	205	52.30	40	54.80	165	51.70	
Current	77	19.60	25	34.20	52	16.30	< 0.001
*Alcohol*							
Non-drinker	104	26.00	14	18.90	90	27.60	
Moderate	90	22.50	11	14.90	79	24.20	
Hazardous/harmful	206	51.50	49	66.20	157	48.20	0.019
*Education*							
School education	170	43.70	37	50.00	133	42.20	
College	158	40.60	28	37.80	130	41.30	
Degree	61	15.70	9	12.20	52	16.50	0.422
*Annual household income*							
< £18,000	138	38.70	36	56.30	102	34.80	
£18,000–£34,999	103	28.90	13	20.30	90	30.70	
> £35,000	116	32.50	15	23.40	101	34.50	0.006
*Marital status*							
Single (never married)	47	11.70	11	14.70	36	11.00	
Currently in relationship	280	69.70	38	50.70	242	74.00	
No longer with spouse	75	18.70	26	34.00	49	15.00	< 0.001

	*N*	Mean (SD)**	*N*	Mean (SD)**	*N*	Mean (SD)**	*p* value
Age at baseline	403	58.4 (9.6)	77	62.86 (11.25)	326	57.39 (8.91)	< 0.001
Body mass index	272	26.4 (4.9)	46	24.33 (4.76)	226	26.88 (4.87)	0.001
*EEAA*	408	− 0.03 (5.78)	77	1.68 (6.52)	331	− 0.42 (5.53)	0.004
*IEAA*	408	− 0.07 (4.37)	77	0.36 (4.34)	331	− 0.17 (4.38)	0.333
*IEAAHannum*	408	− 0.01 (3.94)	77	1.10 (4.52)	331	− 0.27 (3.76)	0.006
*AgeAccelGrim*	408	− 0.10 (5.61)	77	3.16 (5.37)	331	− 0.86 (5.40)	< 0.001
*AgeAccelPheno*	408	− 0.12 (6.57)	77	2.00 (7.04)	331	− 0.62 (6.36)	0.002
*ZhangScore*	408	− 2.20 (0.28)	77	− 2.15 (0.29)	331	− 2.21 (0.27)	0.096

EEAA, extrinsic epigenetic age acceleration; IEAA, intrinsic epigenetic age acceleration, TNM, Tumour, Node, Metastasis. *P* value for difference based on the chi-squared test (categorical) and one-way ANOVA (continuous). * Based on the Adult Comorbidity Evaluation-27 (ACE-27). **For the epigenetic clock measures (IEAA, IEAAHannum, EEAA, AgeAccelPheno and AgeAccelGrim), mean values represent the difference in chronological age and age predicted by the clock, e.g. a mean value of 1.68 indicates that, on average, people who had died at 3 years were predicted to be 1.68 years older than their chronological age at baseline based on their epigenome. A mean age value of − 0.42 indicates that people who were still alive at 3 years were predicted to be, on average, 0.42 years younger than their chronological age. The mortality risk score (ZhangScore), values represent methylation values (rather than years)

### Pairwise correlations between epigenetic markers

**Fig. 2:**
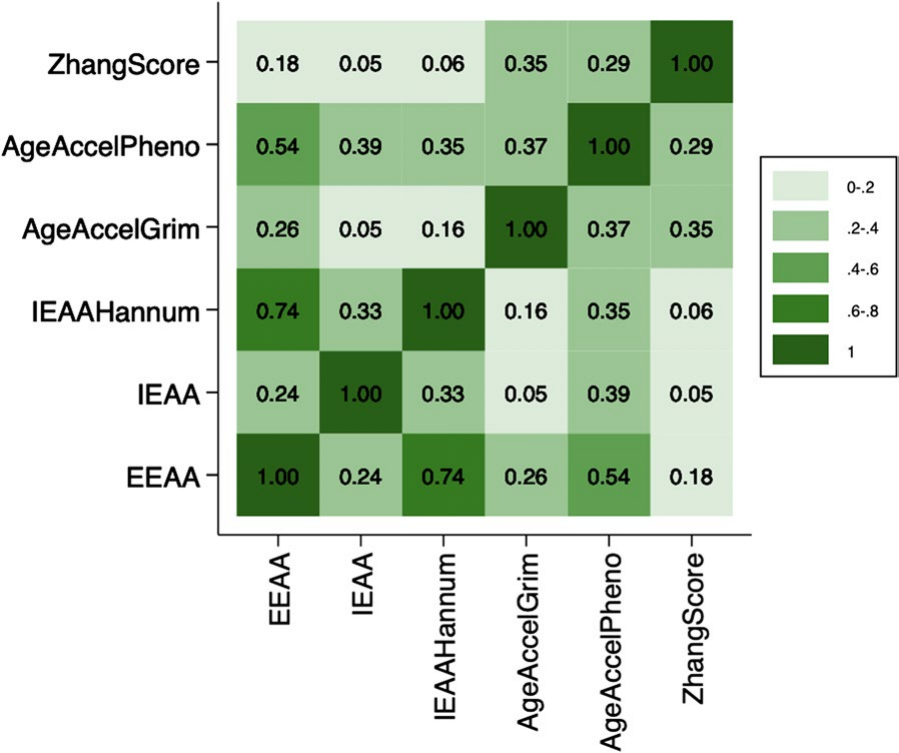
Pairwise correlations between measures of epigenetic age acceleration and the mortality risk score. EEAA, extrinsic epigenetic age acceleration; IEAA, intrinsic epigenetic age

The strongest correlation was between *EEAA* and *IEAAHannum* (0.74) while the weakest was between IEAA and both *AgeAccelGrim* and *ZhangScore* (0.05) (Fig. 2).

### Association of DNAm-based biological age with survival

**Fig. 3:**
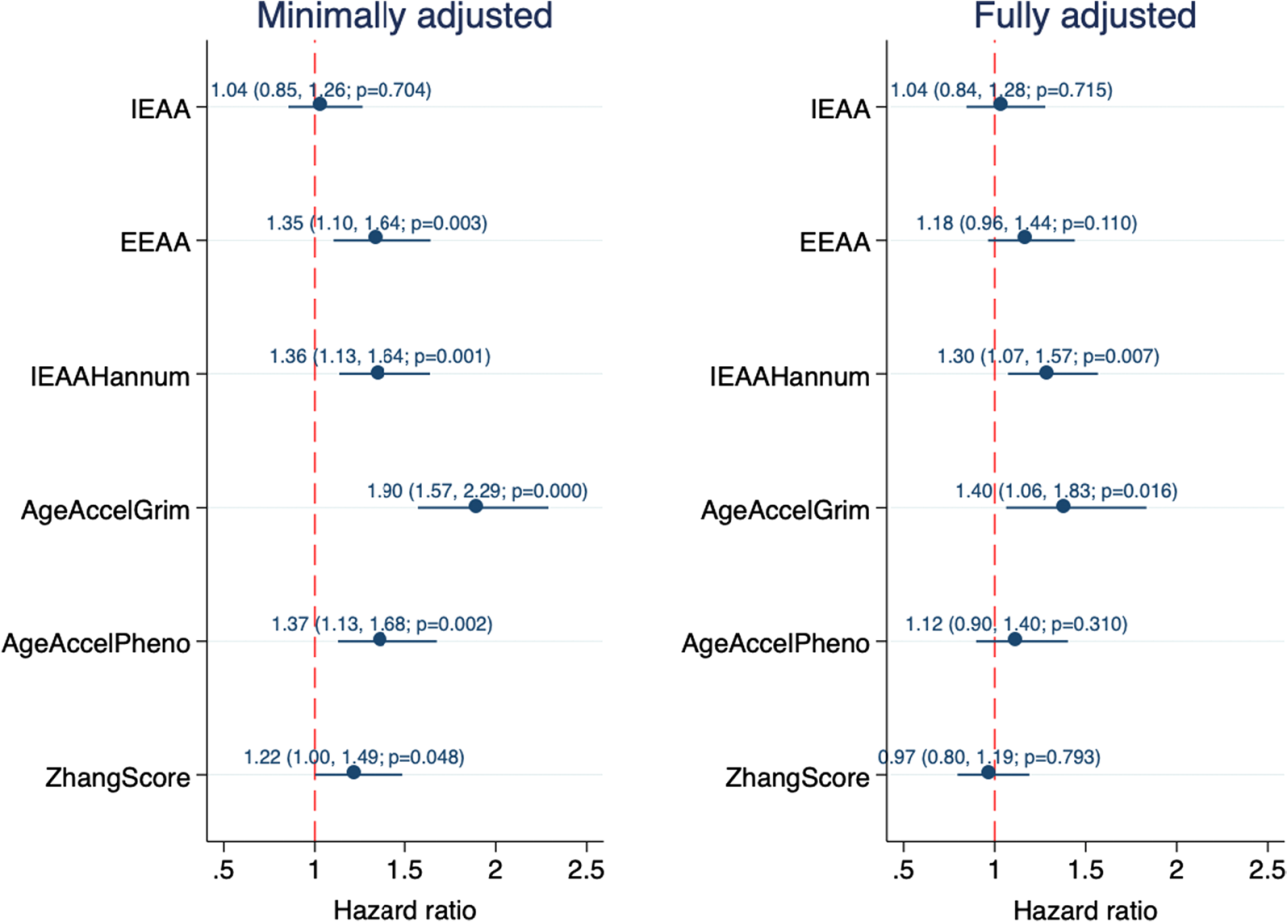
Association of epigenetic age acceleration measures with mortality risk (*n* = 408). EEAA, extrinsic epigenetic age acceleration; IEAA, intrinsic epigenetic age acceleration. Minimally adjusted model included sex (and age for ZhangScores); fully adjusted model included tumour stage, HPV status, comorbidity, BMI, education, income, marital status, smoking status and alcohol consumption

The results of the minimally adjusted and fully adjusted Cox regression analyses on imputed data (*n* = 408) are illustrated in Fig. 3. An overview of all the model outputs is provided in the Supplementary Material (Additional file 1: Table S3).

In the basic model, all the EAA measures except *IEAA* were associated with survival (Fig. 3). The reported associations were in the expected directions, i.e. higher values of EAA were associated with higher mortality risk. HRs ranged from 1.22 (95% CI 1.00, 1.49; *p* = 0.048) for *ZhangScore* to 1.90 (95% CI 1.57, 2.29; *p* = 2.27 × 10^–11^) for *AgeAccelGrim*, where HRs represent the difference in mortality risk per SD unit increase in the epigenetic marker. Associations of *EEAA and ZhangScore* with survival attenuated following adjustment for clinical and socioeconomic factors. In the fully adjusted model, which also adjusted for smoking and alcohol consumption, SD increases in *IEAAHannum* and *AgeAccelGrim* were associated with 30% and 40% increased mortality risks, respectively (HRs 1.30 [95% CI 1.07, 1.57; *p* = 0.007] and 1.40 [95% CI 1.06, 1.83; *p* = 0.016]) (Fig. 3).

In the complete case analysis (*n* = 225; 49 deaths), the results of the minimally adjusted model were broadly comparable (Additional file 1: Table S4) but *IEAAHannum* was not robust to adjustment for socioeconomic factors and the association of *AgeAccelGrim* with survival attenuated following adjustment for smoking and alcohol intake.

Using the continuous measure of alcohol intake (rather than categories) resulted in very similar effect estimates for *IEAA, AgeAccelGrim*, *AgeAccelPheno* and *ZhangScore* in the imputed analysis (Additional file 1: Table S5). The strength of the evidence linking *IEAAHannum* with mortality risk was lower when alcohol units were used (HR 1.22 [0.99, 1.50]; *p* = 0.066). There was some evidence that *EEAA* was associated with mortality risk (HR 1.34 [1.10, 1.62]; *p* = 0.003). The results of the complete case analysis were comparable to those obtained when categories of alcohol exposure were used in model 4 (Additional file 1: Table S5).

The results of the sensitivity analysis, where we included chronological age as a covariate in the epigenetic age models, are presented in Additional file 1: Table S3 (imputed) and Table S4 (complete case). On adjusting for age, the associations of *AgeAccelGrim* and *IEAAHannum* with survival remained in the imputed analysis (fully adjusted HRs 1.50 [1.14, 1.97; *p* = 0.004] and 1.22 [1.00, 1.49; *p* = 0.052), respectively). The strength of the evidence associating these measures with survival was reduced in the complete case analysis, although effect estimates were similar (fully adjusted HRs 1.42 [0.94, 2.14; *p* = 0.095] and 1.23 [0.88, 1.72; *p* = 0.234), respectively)”.

### Examination of the predictive utility of epigenetic markers at 3 years

Table 3 shows the performance measures for the fitted models. The AIC values for the clinical + *IEAA*, clinical + *IEAAHannum* and clinical + *AgeAccelGrim* models were lower than that of the standard clinical model. Two models are generally considered equivalent if the difference in AICs is less than two [55]. On this basis, all three of these models had a better overall fit compared to the standard clinical model. C-statistics ranged from 0.75 (clinical model) to 0.78 (clinical + *AgeAccelGrim* model), but confidence intervals overlapped.

**Table 3:** Measures of model performance for survival prediction

Model	AIC	*C-*statistic (95% CI)
Clinical	486.93	0.75 (0.70, 0.80)
Clinical + *EEAA*	483.36	0.76 (0.71, 0.81)
Clinical + *IEAA*	488.14	0.76 (0.71, 0.81)
Clinical + *IEAAHannum*	480.10	0.77 (0.72, 0.82)
Clinical + *AgeAccelGrim*	473.14	0.78 (0.73, 0.83)
Clinical + *AgeAccelPheno*	485.52	0.76 (0.71, 0.81)
Clinical + *ZhangScore*	488.72	0.75 (0.70, 0.80)

AgeAccelGrim, age acceleration based on DNAmGrimAge; AgeAccelPheno; age acceleration based on PhenoAge; AIC, Akaike information criterion; C-statistic, Harrell’s concordance statistic; EEAA, extrinsic epigenetic age acceleration; IEAA, intrinsic epigenetic age acceleration; ZhangScore, DNA methylation score based on CpG sites found to be associated with mortality risk; 95% CI, 95% confidence interval

When we looked at the effect of adding two of the EAA measures to the clinical model (Additional file 1: Table S6), the *clinical* + *IEAAHannum* + *AgeAccelGrim* had a lower AIC than the clinical + *AgeAccelGrim* model, indicating a better fit to the data, however the C-statistic was not improved compared to the simpler model, indicating that the discriminative ability of the model was no better.

**Fig. 4:**
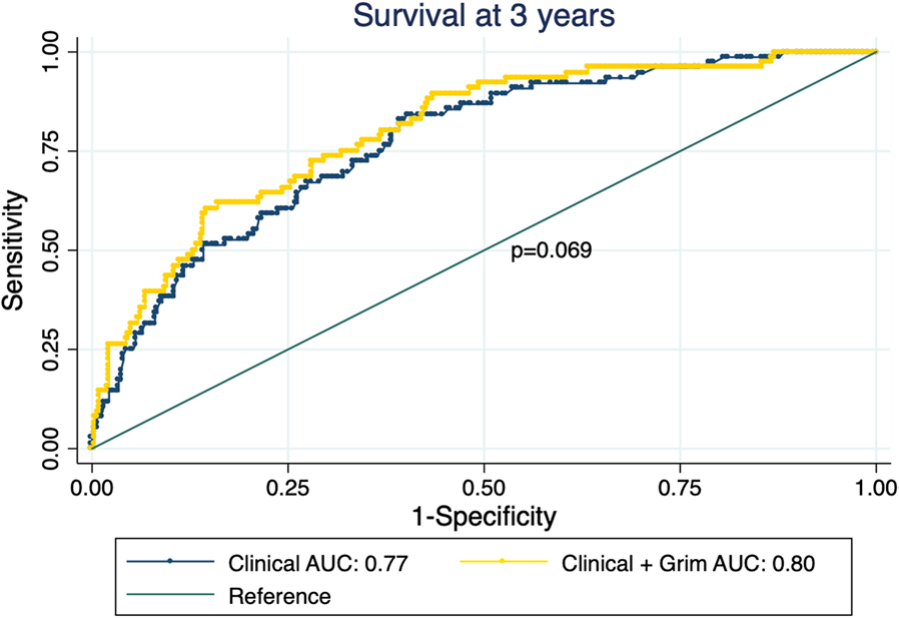
Independent contribution of AgeAccelGrim to prognosis beyond clinical factors. AUC, area under the Roc curve

Given that the clinical + *AgeAccelGrim* model showed the strongest association in the Cox analysis and yielded the highest discrimination, we examined whether this model provided improved prediction at 3 years (*n* = 72 deaths) compared to a standard clinical model including age, sex, TNM stage, HPV and comorbidity, by comparing AUC values. There was weak evidence to suggest the clinical + *AgeAccelGrim* model had superior predictive performance compared to the clinical model (clinical AUC: 0.77, clinical + *AgeAccelGrim* AUC: 0.80; *p* value for difference = 0.069) (Fig. 4). The bootstrap optimism corrected AUC values showed a small reduction in performance compared with the original model (optimism-adjusted AUCs of 0.74 and 0.77 for clinical and clinical + *AgeAccelGrim* models, respectively).

The optimism-adjusted c-slope (uniform shrinkage factor) for the *clinical* + *AgeAccelGrim* model was 0.83, indicating some overfitting. The original predictor effects were adjusted by this value [56] (Table 4). In the adjusted model, each SD unit increase in *AgeAccelGrim* was associated with a 1.5-fold increased risk of death at 3 years (optimism-adjusted HR: 1.54, 95% CI 1.2, 1.92; *p* ≤ 0.001).

**Table 4:** Estimated coefficients (uncorrected and corrected) for the clinical + AgeAccelGrim model

	Original model			Final model after adjustment for overfitting		
		95% CI			95% CI	
Variable	*β*	ll	ul	*β*	ll	ul
Age	0.05	0.02	0.07	0.04	0.02	0.06
*Sex*						
Female	0.42	− 0.14	0.99	0.35	− 0.12	0.82
*Tumour stage*						
II	0.64	− 1.56	2.85	0.53	− 1.29	2.36
III	1.65	− 0.38	3.69	1.37	− 0.32	3.06
IV	1.85	− 0.14	3.84	1.54	− 0.12	3.19
*HPV status*						
Positive	− 0.95	− 1.47	− 0.44	− 0.79	− 1.22	− 0.36
Comorbidity*						
Mild	0.33	− 0.23	0.90	0.28	− 0.19	0.75
Moderate/severe	0.24	− 0.38	0.85	0.20	− 0.31	0.70
AgeAccelGrim	0.52	0.26	0.78	0.43	0.22	0.65

Regression coefficients (ß) and 95% confidence intervals (CI) for 3-year overall survival

Hazard ratios can be obtained by exponentiating model estimates

Smoking has been shown to be independently predictive of mortality in H&N5000 [17]. The reduced effect estimate observed between *AgeAccelGrim* and mortality with adjustment for smoking status suggests that the enhanced prognostic ability gained from adding *AgeAccelGrim* to the clinical model could be due to the inclusion of a smoking predictor [35]. We conducted an additional sensitivity analysis (Additional file 2: Fig. S1) whereby we compared the prognostic ability of the following models: (1) clinical + *AgeAccelGrim*; (2) clinical + self-reported smoking; and (3) clinical + *DNAmpackyears*, the DNAm-based surrogate biomarker for pack-years of smoking used to derive GrimAge (*n* = 384 participants with smoking data available; no. deaths = 72). At 3 years, there was a suggestion that the clinical + *AgeAccelGrim* model had better discrimination (AUC value of 0.80 [95% 95% CI 0.74, 0.85]) than the clinical models including both self-reported smoking (AUC = 0.77 [95% CI 0.71, 0.83]) and a DNAm surrogate for pack-years (AUC = 0.78 [0.72, 0.83]), although there was limited evidence of a difference in AUCs based on chi-squared tests (*p* = 0.148).

## Discussion

In this study of 408 OPC cases with a median of 5 years of follow-up, we demonstrate that epigenetic markers derived from blood are associated with increased risk of all-cause mortality and these associations are independent of established mortality risk factors. In particular, *AgeAccelGrim,* an “extrinsic” age acceleration measure which captures exogenous lifestyle factors and extracellular changes related to ageing, had the strongest effect estimate, with each SD increase in EAA resulting in a 40% increase in risk of death in the fully adjusted model (HR 1.40; 95% CI 1.06, 1.83; *p* = 0.016). *IEEAHannum*, an “intrinsic” measure of EAA, was also associated with mortality risk, but to a lesser extent. The addition of *AgeAccelGrim* to the clinical model showed marginal improvement in mortality risk prediction at 3 years (Clinical AUC: 0.77, Clinical + *AgeAccelGrim* AUC: 0.80; *p* = 0.069). Our findings support the literature which suggests that age acceleration as measured by GrimAge is a better predictor of mortality risk in healthy populations compared to first-generation DNAm-based predictors (i.e. Horvath and Hannum’s clocks) [35].

It is unclear why some epigenetic ageing measures can predict mortality risk better than others in this population. The DNAm clocks used to derive these measures reflect different aspects of cellular processes and exogenous factors (i.e. lifestyle factors). Smoking has been shown to be independently predictive of mortality among HNC cases [17], therefore it is possible that the relatively strong association of *AgeAccelGrim* with mortality risk may be explained by the inclusion of the surrogate measure for smoking in the GrimAge biomarker. When we compared the prognostic performance of the clinical + *AgeAccelGrim* model with clinical models including both self-reported smoking and the DNAm surrogate biomarker for pack-years of smoking, clinical + *AgeAccelGrim* had better discrimination. While the difference in model performance was modest, it nonetheless suggests that the methylation-based measure of smoking provides a better indicator with less misclassification than self-report. Moreover, the prognostic utility of *AgeAccelGrim* does not appear to be solely driven by the inclusion of the DNAm-based biomarker for smoking. GrimAge is also trained on a set of proteins known to be associated with mortality [35]. One of these, Plasminogen activator inhibitor 1 (PAI-1), is overexpressed in a variety of tumours and is a strong predictor of poor clinical outcomes [57–59]. Another, growth differentiation factor 15 (GDF15) is involved in the pathogenesis of oral squamous cell carcinoma (OSCC) [60–62]. Further studies are needed to examine whether these factors may be contributing to the prognostic utility of GrimAge.

Hannum and Horvath’s clocks were built using similar regression techniques and show moderate correlation, yet, in our analysis, only *IEAAHannum* was associated with survival. This finding is consistent with previous work [25]*.* It is possible that, because the Hannum predictor was developed and validated in blood samples—the tissue type used in our analysis—it may be better able to capture cell-intrinsic processes in blood compared with a predictor that was developed across multiple tissue, i.e. Horvath’s predictor.

Our investigation has several strengths including the relatively long follow-up period, the fact that individuals were sampled at the time of diagnosis and that DNAm was assayed in the same laboratory. We were also able to account for a range of factors which are known to influence both DNAm and HNC risk [63, 64] and missing covariate data were imputed to minimise possible biases [65, 66].

Our study has several limitations. First, the sample size for our analysis was relatively small and we were unable to identify independent prospective datasets to validate our findings. This limits the translation impact of our work. To mitigate this, we obtained estimates of a uniform shrinkage factor and multiplied this by the original β-coefficients from the fitted model to obtain optimism-adjusted coefficients. Second, various unmeasured confounders may influence the outcome of these age predictors, including genetic and environmental factors. While we found that the associations of GrimAge and IEAAHannum persisted after controlling for smoking and alcohol intake in our primary analyses, residual confounding is likely to be present. This is especially likely since we used categories of exposure which were derived via participants’ self-report, which is prone to recall bias and/or misreporting. We conducted sensitivity analyses to evaluate residual confounding by alcohol based on a continuous variable of units/week and found that the effect estimates for *AgeAccelGrim* were comparable to those of our primary analysis (HR 1.40 [1.05, 1.85]; *p* = 0.020 in the model that included units of alcohol consumed vs 1.40 [1.06, 1.83]; *p* = 0.016 in the model that included categories of alcohol consumption). The association of *IEAAHannum* with mortality remained when alcohol units were used but the HR was lower (HR 1.22 [0.99, 1.50]; *p* = 0.066 vs 1.30 [1.07, 1.57]; *p* = 0.007 for the model that included alcohol categories). While we were unable to derive a continuous measure for lifetime smoking, we utilised a DNAm-derived measure of pack-years of smoking in our sensitivity analysis for GrimAge. We found that the addition of *AgeAccelGrim* to a clinical model that included age, tumour stage and HPV status had slightly better discrimination (AUC = 0.80) compared to a clinical model that additionally included the DNAm surrogate marker for smoking (AUC = 0.78). Genome-wide DNA methylation (DNAm) profiling has allowed for the development of molecular predictors for a multitude of traits and diseases, including smoking and alcohol intake [60]. Future studies could implement the use of other methylation scores to index these variables [63, 64]. Third, there is a disparity in coverage between Illumina 450 K and EPIC platforms meaning that 17 of the 353 CpGs (4.8%), and 6 of the 71 CpGs (8.5%) necessary to calculate epigenetic age via the Horvath and Hannum methods, respectively, were missing [67]. Similarly, two of the CpGs included in the DNAm risk score for mortality were missing from the DNA methylation dataset for the same reason. Previous work suggests that the lack of the clock-CpGs on the EPIC array does not undermine the utility of the epigenetic age predictors [68]. Fourth, we did not account for multiple testing, although evidence of correlation between some of the epigenetic measures suggests that correction may not have been appropriate. Finally, it was not possible to examine cancer-specific mortality.

## Conclusion

DNAm-based estimators of ageing could provide prognostic utility in people with OPC, above established prognostic factors, though the mechanisms of association are currently unclear. That an accurate, easy-to-measure biomarker could serve as a better predictor of mortality risk is important as it could aid treatment planning and improve patient stratification in study design. These findings should be investigated in further, independent samples.

## Supplementary Information

**Additional file 1. Table S1**. Proportion of missing data, N = 408;** Table S2**. Baseline descriptives of participants included in the complete case analysis (n = 225);** Table S3**. Association of DNA Methylation-Based predictors of Ageing with overall-survival based on imputed data (n = 408);** Table S4**. Results of the complete case cox regression analysis (n = 225);** Table S5**. Results of the sensitivity analyses, adjusting for units of alcohol consumed per week (Model 4);** Table S6**. The impact of including two epigenetic age acceleration measures on model fit and discrimination.

**Additional file 2. Supplementary Figure 1**. A comparison of the area under the ROC curves (AUC) obtained for the models included in the sensitivity analyses (n = 384).

**Additional file 3.** Additional methods.

## Data Availability

The datasets used in this analysis are available from the Head and Neck 5000 study upon submission of a research proposal. If you would like to access this resource, please contact the Head and Neck 5000 Executive on headandneck5000@uhbristol.nhs.uk. The study website http://www.headandneck5000.org.uk/ describes the resource and the types of data available.

## Abbreviations

AIC: Akaike information criterion
AUC: Area under the receiver operating characteristic curve
BIC: Bayesian information criterion
CI: Confidence interval
CpG: Cytosine–phosphate–guanine site
EAA: Epigenetic age acceleration
HNC: Head and neck cancer
HPV: Human papillomavirus
HR: Hazard ratio
IQR: Inter-quartile range
MAR: Missing at random
OPC: Oropharyngeal cancer
SD: Standard deviation
