# Supplementary material for: Epigenetic biomarkers of ageing are predictive of mortality risk in a longitudinal clinical cohort of individuals diagnosed with oropharyngeal cancer
Source: Clin Epigenetics. 2022 Jan 3;14:1. doi: 10.1186/s13148-021-01220-4 (PMC8725548; doi:10.1186/s13148-021-01220-4)
Supplement: Supplementary file 1 — Additional file 1. Table S1. Proportion of missing data, N = 408; Table S2. Baseline descriptives of participants included in the complete case analysis (n = 225); Table S3. Association of DNA Methylation-Based predictors of Ageing with overall-survival based on imputed data (n = 408); Table S4. Results of the complete case cox regression analysis (n = 225); Table S5. Results of the sensitivity analyses, adjusting for units of alcohol consumed per week (Model 4); Table S6. The impact of including two epigenetic age acceleration measures on model fit and discrimination. [file 13148_2021_1220_MOESM1_ESM.docx]

*Table S1: Proportion of missing data, N=408*

| ***Variable*** | ***Missing*** | ***% Missing*** |
| --- | --- | --- |
| Age | 5 | 1.23 |
| Sex | 0 | 0 |
| Ethnicity | 10 | 2.45 |
| TNM stage | 0 | 0 |
| HPV status | 0 | 0 |
| Comorbidity | 3 | 0.74 |
| BMI | 136 | 33.33 |
| Annual household income | 51 | 12.5 |
| Education | 19 | 4.66 |
| Marital status | 6 | 1.47 |
| Smoking status | 16 | 3.92 |
| Alcohol intake (units/week*) | 8 | 1.96 |

*Abbreviations: BMI, body mass index; TNM, tumour (T), node (N), metastasis (M) staging*

* 1 unit = 10ml/8g of pure ethanol*.*

*Supplementary table 2: Baseline descriptives of participants included in the complete case analysis (n=225)*

|  | Dead at 3 years n=37 | | Alive at 3 years n=188 | |  |
| --- | --- | --- | --- | --- | --- |
| Characteristic | N | % | N | % | p-value |
| **Gender** |  |  |  |  |  |
| Male | 30 | 81.1% | 147 | 78.2% |  |
| Female | 7 | 18.9% | 41 | 21.8% | 0.695 |
| **TNM stage group** |  |  |  |  |  |
| I | 1 | 2.7% | 11 | 5.9% |  |
| II | 3 | 8.1% | 19 | 10.1% |  |
| III | 3 | 8.1% | 21 | 11.2% |  |
| IV | 30 | 81.1% | 137 | 72.9% | 0.740 |
| **HPV status** |  |  |  |  |  |
| Negative | 22 | 59.5% | 44 | 23.4% |  |
| Positive | 15 | 40.5% | 144 | 76.6% | <0.001 |
| **Comorbidity status*** |  |  |  |  |  |
| None | 18 | 48.6% | 116 | 61.7% |  |
| Mild | 14 | 37.8% | 45 | 23.9% |  |
| Moderate/severe | 5 | 13.5% | 27 | 14.4% | 0.204 |
| **Smoking** |  |  |  |  |  |
| Never | 2 | 5.4% | 70 | 37.2% |  |
| Former | 20 | 54.1% | 97 | 51.6% |  |
| Current | 15 | 40.5% | 21 | 11.2% | <0.001 |
| **Alcohol** |  |  |  |  |  |
| Non-drinker | 7 | 18.9% | 52 | 27.7% |  |
| Moderate | 5 | 13.5% | 49 | 26.1% |  |
| Hazardous/harmful | 25 | 67.6% | 87 | 46.3% | 0.057 |
| **Education** |  |  |  |  |  |
| School education | 18 | 48.6% | 83 | 44.1% |  |
| College | 14 | 37.8% | 78 | 41.5% |  |
| Degree | 5 | 13.5% | 27 | 14.4% | 0.880 |
| **Annual household income** |  |  |  |  |  |
| <£18,000 | 23 | 62.2% | 65 | 34.6% |  |
| £18000-£34,999 | 5 | 13.5% | 62 | 33.0% |  |
| >£35,000 | 9 | 24.3% | 61 | 32.4% | 0.005 |
| **Marital status** |  |  |  |  |  |
| single (never married) | 7 | 18.9% | 16 | 8.5% |  |
| currently in relationship | 16 | 43.2% | 142 | 75.5% |  |
| No longer with spouse | 14 | 37.8% | 30 | 16.0% | <0.001 |
|  | N | mean (SD)** | N | mean (SD)** | p-value |
| Age at baseline | 37 | 62.76 (12.12) | 188 | 56.93 (8.90) | 0.001 |
| Body mass index | 37 | 23.83 (4.89) | 188 | 26.70 (4.86) | 0.001 |
| *IEAA* | 37 | 1.38 (6.52) | 188 | -0.44 (5.64) | 0.082 |
| *EEAA* | 37 | 0.74 (3.99) | 188 | -0.19 (4.34) | 0.226 |
| *IEAAHannum* | 37 | 1.29 (4.69) | 188 | -0.33 (3.84) | 0.025 |
| *AgeAccelPheno* | 37 | 4.17 (5.42) | 188 | -1.14 (5.41) | <0.001 |
| *AgeAccelGrim* | 37 | 3.01 (7.30) | 188 | -0. (6.26) | 0.002 |

*Abbreviations: EEAA, extrinsic epigenetic age acceleration; IEAA, intrinsic epigenetic age acceleration. P-value for difference based on the Chi-Square test (categorical) and one-way ANOVA (continuous). *Based on the Adult Comorbidity Evaluation-27 (ACE-27). ** values for raw epigenetic age acceleration measures.*

*Supplementary Table 3: Association of DNA Methylation-Based predictors of Ageing with overall-survival based on imputed data (n=408).*

|  | **Primary analysis** | | | |  | **Adjusting for chronological age** | | | |
| --- | --- | --- | --- | --- | --- | --- | --- | --- | --- |
| Epigenetic clock | HR | Lower CI | Upper CI | *p*-value |  | HR | Lower CI | Upper CI | *p*-value |
| Basic model | | | | | | | | | |
| *IEAA* | 1.04 | 0.85 | 1.26 | 0.704 |  | 1.06 | 0.88 | 1.29 | 0.532 |
| *EEAA* | 1.35 | 1.10 | 1.64 | 0.003 |  | 1.35 | 1.13 | 1.62 | 0.001 |
| *IEAAHannum* | 1.36 | 1.13 | 1.64 | 0.001 |  | 1.36 | 1.12 | 1.65 | 0.002 |
| *AgeAccelGrim* | 1.90 | 1.57 | 2.29 | 2.27 x 10^-11^ |  | 1.99 | 1.63 | 2.41 | 5.86 x 10^-12^ |
| *AgeAccelPheno* | 1.37 | 1.13 | 1.68 | 0.002 |  | 1.35 | 1.11 | 1.65 | 0.003 |
| *ZhangScore* | 1.22 | 1.00 | 1.49 | 0.048 |  | . | . | . | . |
| Clinical model | | | | | | | | | |
| *IEAA* | 1.03 | 0.85 | 1.26 | 0.757 |  | 1.03 | 0.85 | 1.26 | 0.738 |
| *EEAA* | 1.23 | 1.00 | 1.50 | 0.049 |  | 1.36 | 1.13 | 1.65 | 0.001 |
| *IEAAHannum* | 1.34 | 1.11 | 1.63 | 0.003 |  | 1.27 | 1.04 | 1.55 | 0.021 |
| *AgeAccelGrim* | 1.60 | 1.30 | 1.98 | 1.39x10^-05^ |  | 1.72 | 1.39 | 2.15 | 1.03 x 10^-06^ |
| *AgeAccelPheno* | 1.19 | 0.97 | 1.47 | 0.102 |  | 1.20 | 0.98 | 1.48 | 0.082 |
| *ZhangScore* | 1.10 | 0.91 | 1.33 | 0.327 |  | . | . | . | . |
| Socioeconomic model | | | | | | | | | |
| *IEAA* | 1.03 | 0.84 | 1.26 | 0.790 |  | 1.03 | 0.85 | 1.26 | 0.746 |
| *EEAA* | 1.21 | 0.99 | 1.49 | 0.060 |  | 1.35 | 1.12 | 1.64 | 0.002 |
| *IEAAHannum* | 1.33 | 1.10 | 1.62 | 0.003 |  | 1.26 | 1.03 | 1.54 | 0.025 |
| *AgeAccelGrim* | 1.52 | 1.22 | 1.89 | 2.03 x10^-04^ |  | 1.65 | 1.32 | 2.08 | 1.41 X 10^-05^ |
| *AgeAccelPheno* | 1.22 | 0.98 | 1.51 | 0.078 |  | 1.23 | 0.99 | 1.52 | 0.064 |
| *ZhangScore* | 1.08 | 0.89 | 1.31 | 0.458 |  | . | . | . | . |
| Behavioural model | | | | | | | | | |
| *IEAA* | 1.04 | 0.84 | 1.28 | 0.715 |  | 1.04 | 0.85 | 1.28 | 0.696 |
| *EEAA* | 1.18 | 0.96 | 1.44 | 0.110 |  | 1.31 | 1.09 | 1.57 | 0.004 |
| *IEAAHannum* | 1.30 | 1.07 | 1.57 | 0.007 |  | 1.22 | 1.00 | 1.49 | 0.052 |
| *AgeAccelGrim* | 1.40 | 1.06 | 1.83 | 0.016 |  | 1.50 | 1.14 | 1.97 | 0.004 |
| *AgeAccelPheno* | 1.12 | 0.90 | 1.40 | 0.310 |  | 1.11 | 0.89 | 1.39 | 0.361 |
| *ZhangScore* | 0.97 | 0.80 | 1.19 | 0.793 |  | . | . | . | . |

*Abbreviations: EEAA, extrinsic epigenetic age acceleration; IEAA, intrinsic epigenetic age acceleration. In the primary analysis, the “Basic model” adjusted for gender; the” clinical model” additionally adjusted for tumour stage, HPV status, comorbidity and BMI; the “socioeconomic model” additionally adjusted for education, income and marital status; and the “behavioural model” adjusted for smoking status and alcohol consumption. As a sensitivity analysis, cox models were additionally adjusted for chronological age (all models). N.B. the ZhangScore model was adjusted for chronological age in the primary analysis (since age was not used in the generation of this measure). There were 105 deaths* during follow-up (median=5.3 years, IQR 4.9 to 6.0).

*Supplementary table 4: Results of the complete case cox regression analysis (n=225).*

|  | **Primary analysis** | | | |  | **Adjusting for chronological age** | | | |
| --- | --- | --- | --- | --- | --- | --- | --- | --- | --- |
| Epigenetic clock | HR | Lower CI | Upper CI | *p*-value |  | HR | Lower CI | Upper CI | *p*-value |
| Basic model | | | | | | | | | |
| *IEAA* | 1.01 | 0.75 | 1.35 | 0.960 |  | 0.99 | 0.75 | 1.31 | 0.951 |
| *EEAA* | 1.41 | 1.04 | 1.91 | 0.026 |  | 1.29 | 0.99 | 1.68 | 0.062 |
| *IEAAHannum* | 1.36 | 1.04 | 1.8 | 0.027 |  | 1.39 | 1.03 | 1.88 | 0.032 |
| *AgeAccelGrim* | 1.96 | 1.52 | 2.53 | 2.8 x10^-07^ |  | 2.06 | 1.57 | 2.70 | 2.05 x 10^-07^ |
| *AgeAccelPheno* | 1.53 | 1.14 | 2.06 | 5.0 x10^-03^ |  | 1.48 | 1.10 | 1.99 | 0.009 |
| *ZhangScore* | 1.14 | 0.83 | 1.55 | 0.413 |  | . | . | . | . |
| *Clinical model* | | | | | | | | | |
| *IEAA* | 0.92 | 0.68 | 1.26 | 0.620 |  | 0.93 | 0.68 | 1.26 | 0.625 |
| *EEAA* | 1.27 | 0.93 | 1.74 | 0.130 |  | 1.35 | 1.02 | 1.79 | 0.034 |
| *IEAAHannum* | 1.34 | 1.01 | 1.77 | 0.042 |  | 1.37 | 0.99 | 1.89 | 0.059 |
| *AgeAccelGrim* | 1.66 | 1.23 | 2.24 | 8.2 x10^-04^ |  | 1.84 | 1.34 | 2.51 | 1.38 x 10^-04^ |
| *AgeAccelPheno* | 1.35 | 0.98 | 1.85 | 0.066 |  | 1.43 | 1.04 | 1.98 | 0.030 |
| *ZhangScore* | 1.03 | 0.76 | 1.40 | 0.848 |  | . | . | . | . |
| *Socioeconomic model* | | | | | | | | | |
| *IEAA* | 0.93 | 0.68 | 1.27 | 0.640 |  | 0.93 | 0.68 | 1.27 | 0.653 |
| *EEAA* | 1.14 | 0.82 | 1.58 | 0.440 |  | 1.32 | 0.98 | 1.78 | 0.069 |
| *IEAAHannum* | 1.29 | 0.95 | 1.74 | 0.100 |  | 1.25 | 0.88 | 1.76 | 0.211 |
| *AgeAccelGrim* | 1.45 | 1.04 | 2.02 | 0.027 |  | 1.64 | 1.16 | 2.30 | 0.005 |
| *AgeAccelPheno* | 1.23 | 0.89 | 1.72 | 0.210 |  | 1.28 | 0.92 | 1.78 | 0.147 |
| *ZhangScore* | 0.97 | 0.71 | 1.34 | 0.859 |  | . | . | . | . |
| *Behavioural model* | | | | | | | | | |
| *IEAA* | 0.90 | 0.66 | 1.24 | 0.520 |  | 0.90 | 0.66 | 1.22 | 0.483 |
| *EEAA* | 1.12 | 0.81 | 1.55 | 0.490 |  | 1.28 | 0.95 | 1.72 | 0.103 |
| *IEAAHannum* | 1.25 | 0.92 | 1.68 | 0.150 |  | 1.23 | 0.88 | 1.72 | 0.234 |
| *AgeAccelGrim* | 1.24 | 0.83 | 1.85 | 0.290 |  | 1.42 | 0.94 | 2.14 | 0.095 |
| *AgeAccelPheno* | 1.22 | 0.87 | 1.7 | 0.260 |  | 1.25 | 0.88 | 1.76 | 0.209 |
| *ZhangScore* | 0.92 | 0.67 | 1.28 | 0.629 |  | . | . | . | . |

*Abbreviations: EEAA, extrinsic epigenetic age acceleration; IEAA, intrinsic epigenetic age acceleration. The “Basic model” adjusted for gender; the” clinical model” additionally adjusted for tumour stage, HPV status, comorbidity and BMI; the “socioeconomic model” additionally adjusted for education, income and marital status; and the “behavioural model” adjusted for smoking status and alcohol consumption. As a sensitivity analysis, cox models were additionally adjusted for chronological age (all models). N.B. the ZhangScore model was adjusted for chronological age in the primary analysis (since age was not used in the generation of this measure). There were 49 deaths during follow-up* (median=5.4 years, IQR 4.9 to 6.0).

*Supplementary table 5: Results of the sensitivity analyses, adjusting for units of alcohol consumed per week (Model 4).*

| **Complete case** | | | | |  | **Imputed analysis** | | | |
| --- | --- | --- | --- | --- | --- | --- | --- | --- | --- |
|  | HR | Lower CI | Upper CI | p-value |  | HR | Lower CI | Upper CI | p-value |
| Behavioural model | | | | | | | | | |
| *IEAA* | 0.90 | 0.66 | 1.23 | 0.516 |  | 1.05 | 0.85 | 1.30 | 0.650 |
| *EEAA* | 1.12 | 0.81 | 1.54 | 0.505 |  | 1.34 | 1.10 | 1.62 | 0.003 |
| *IEAAHannum* | 1.23 | 0.92 | 1.66 | 0.167 |  | 1.22 | 0.99 | 1.50 | 0.060 |
| *AgeAccelGrim* | 1.24 | 0.83 | 1.86 | 0.293 |  | 1.40 | 1.05 | 1.85 | 0.020 |
| *AgeAccelPheno* | 1.20 | 0.85 | 1.68 | 0.303 |  | 1.13 | 0.90 | 1.41 | 0.285 |
| *ZhangScore* | 0.91 | 0.67 | 1.25 | 0.573 |  | 0.97 | 0.79 | 1.19 | 0.763 |

*Abbreviations: EEAA, extrinsic epigenetic age acceleration; IEAA, intrinsic epigenetic age acceleration. The “behavioural model” adjusted for gender, stage, HPV status, comorbidity, BMI, education, income, marital status, smoking status and units of alcohol consumed per week (rather than alcohol categories)..*

*Supplementary Table 6: The impact of including two epigenetic age acceleration measures on model fit and discrimination.*

| **Model** |  |  | **C-stat (95% CI)** | **AIC** |
| --- | --- | --- | --- | --- |
| Single | | | | |
| clinical |  |  | 0.75 (0.70, 0.80) | 486.93 |
| clinical + EEAA | |  | 0.76 (0.71, 0.81) | 483.38 |
| clinical + IEAA | |  | 0.76 (0.71, 0.81) | 488.14 |
| clinical + IEAAhannum | |  | 0.77 (0.72, 0.82) | 480.10 |
| clinical + AgeAccelGrim | |  | 0.78 (0.73, 0.83) | 473.14 |
| clinical + AgeAccelPheno | |  | 0.76 (0.71, 0.81) | 485.52 |
| clinical +AgeAccelZhang | |  | 0.76 (0.70, 0.81) | 487.47 |
| Combined | | | | |
| clinical + EEAA + IEAA | |  | 0.78 (0.73, 0.83) | 485.22 |
| clinical + EEAA + IEAAhannum | | | 0.76 (0.71, 0.82) | 482.09 |
| clinical + EEAA + AgeAccelGrim | | | 0.78 (0.73, 0.83) | 471.45 |
| clinical + EEAA + AgeAccelPheno | | | 0.76 (0.70. 0.81) | 484.81 |
| clinical + EEAA + AgeAccelZhang | | | 0.76 (0.71, 0.81) | 484.92 |
| clinical + IEAA + IEAAhannum | | | 0.77 (0.71, 0.82) | 482.10 |
| clinical + IEAA + AgeAccelGrim | | | 0.78 (0.73, 0.83) | 474.32 |
| clinical + IEAA + AgeAccelZhang | | | 0.76 (0.71, 0.81) | 488.46 |
| clinical + IEAA + AgeAccelPheno | | | 0.76 (0.71, 0.81) | 487.47 |
| clinical + IEAAhannum + AgeAccelGrim | | | 0.78 (0.73, 0.83) | 467.93 |
| clinical + IEAAhannum + AgeAccelPheno | | | 0.77 (0.72, 0.82) | 481.44 |
| clinical + IEAAhannum + AgeAccelZhang | | | 0.77 (0.72, 0.82) | 479.72 |
| clinical + AgeAccelGrim + AgeAccelPheno | | | 0.78 (0.73, 0.83) | 474.63 |
| clinoical + AgeAccelGrim + AgeAccelZhang | | | 0.78 (0.73, 0.83) | 474.73 |
| clinical + AgeAccelPheno + AgeAccelZhang | | | 0.76 (0.71, 0.81) | 486.97 |
