# Supplementary material for: Epigenetic biomarkers of ageing are predictive of mortality risk in a longitudinal clinical cohort of individuals diagnosed with oropharyngeal cancer
Source: Clin Epigenetics. 2022 Jan 3;14:1. doi: 10.1186/s13148-021-01220-4 (PMC8725548; doi:10.1186/s13148-021-01220-4)
Supplement: Supplementary file 2 — Additional file 2. Supplementary Figure 1. A comparison of the area under the ROC curves (AUC) obtained for the models included in the sensitivity analyses (n = 384). [file 13148_2021_1220_MOESM2_ESM.docx]

*Supplementary Figure 1:* *A comparison of the area under the ROC curves (AUC) obtained for the models included in the sensitivity analyses (n=384).*


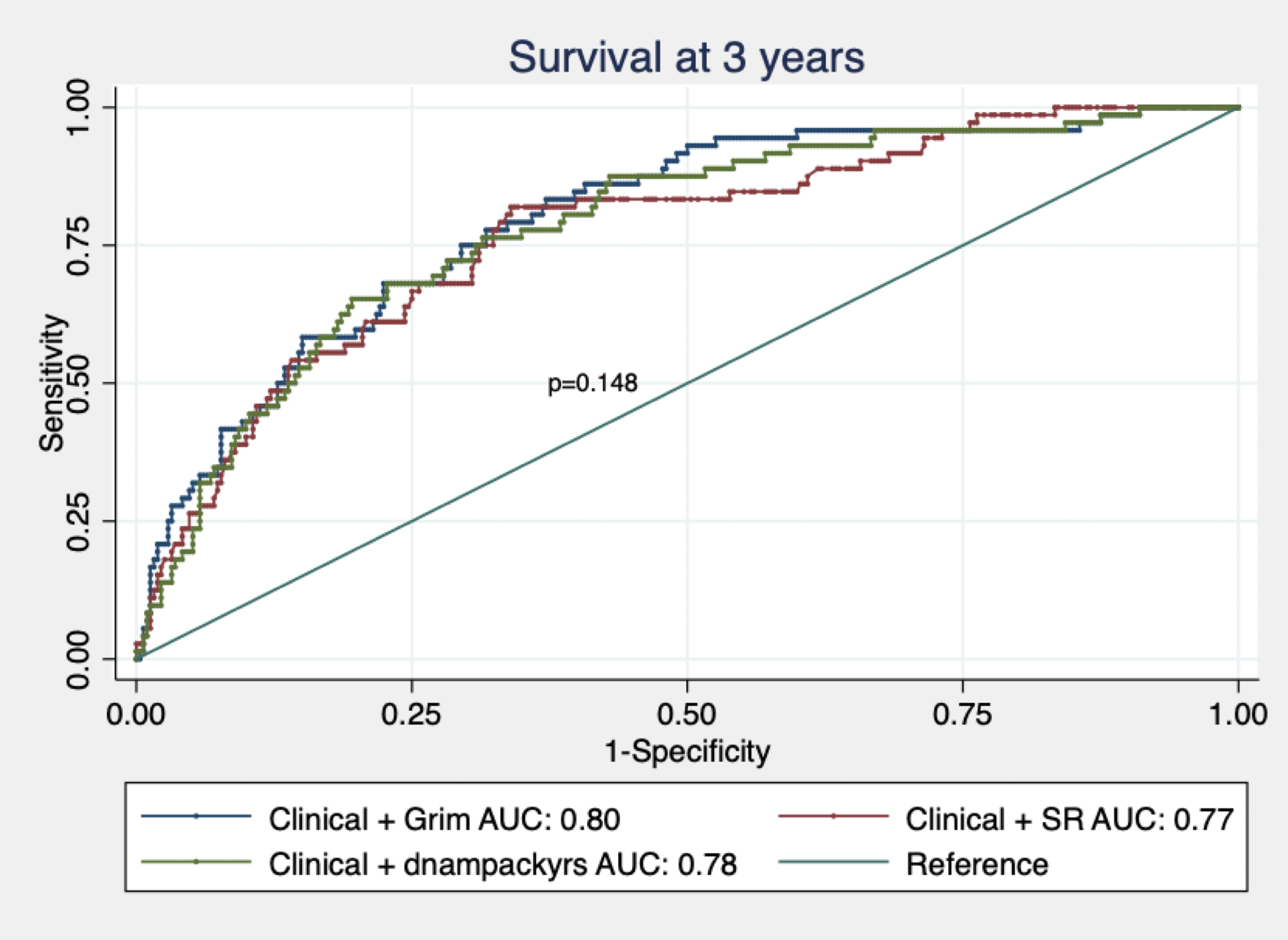


No. deaths at 3-years =72.

Clinical model included age, sex, TNM stage, HPV status and comorbidity. Abbreviations: AUC, area under the receiver operating characteristics curve; DNAmpackyrs, the DNA methylation based biomarker of pack years of smoking used to derive GrimAge, (29); Grim, age acceleration based on the GrimAge measure (29); SR, self-reported smoking status. P-value for difference.
