# Supplementary material for: Epigenetic biomarkers of ageing are predictive of mortality risk in a longitudinal clinical cohort of individuals diagnosed with oropharyngeal cancer
Source: Clin Epigenetics. 2022 Jan 3;14:1. doi: 10.1186/s13148-021-01220-4 (PMC8725548; doi:10.1186/s13148-021-01220-4)
Supplement: Supplementary file 3 — Additional file 3. Additional methods. [file 13148_2021_1220_MOESM3_ESM.docx]

Supplementary Methods

.

*Assessment of smoking and alcohol intake*

Baseline smoking status was based on participants’ self-report. Participants were asked whether they would describe themselves as a “current”, “former” or “never” user of tobacco. The definition of a “never-user”, as outlined in the questionnaire, was someone who had never used tobacco on a regular basis, i.e. one tobacco product per day for a period of one year. By that definition, current smokers were defined as smokers of at least 1 cigarette per day over the course of a year at the time of blood sample collection.

Participants were asked to report the number of alcoholic beverages they consumed on an average week before they became ill, providing information separately for wine (in glasses), spirits (in measures), and beer/larger/cider (in pints). Initially, drinks were converted into grams of ethanol, where 1 UK unit translates to 10ml, or 8g, of pure alcohol. Drinking categories were then created based on UK guidelines: none, moderate, hazardous to harmful. Moderate drinkers included men and women who drink < 14 units/week; hazardous drinkers included men who consumed 14 – 50 units/week and women who consumed 14 – 35 units/week; harmful drinkers included men who consumed > 50 units/week and women who consumed > 35 units/week. Participants were classed as non-drinkers if both drink-days per week was zero and units per week was zero.

*DNA methylation profiling*

The participants were selected for DNA methylation profiling based on clinical ICD-10 coding of OPC. To date, DNA samples isolated from buffy coats have been analysed for 448 participants. Following extraction, DNA was bisulphite-converted using the Zymo EZ DNA Methylation^TM^ kit (Zymo, Irvine, CA, USA). Genome-wide methylation data were generated using the Infinium MethylationEPIC BeadChip (EPIC array) (Illumina, USA) according to the manufacturer protocol. The arrays were scanned using an Illumina iScan (version 2.3). Raw data files (IDAT files) were pre-processed using the R package *meffil* (<https://github.com/perishky/meffil/)> ^1^ to perform quality control (QC) and normalization, where control probes are utilised to separate biological variation from technical variation. Overall, 440/448 samples passed QC (sample exclusions: 2 samples with incorrect sex prediction, 3 samples with sex detection outliers, 1 sample with an outlier in predicted median methylated vs unmethylated signal, 2 duplicate samples) and were normalized. For the samples, the methylation level at each CpG site was calculated as a beta value (*β*), which is the ratio of the methylated probe intensity and the overall intensity and ranges from 0 (no cytosine methylation) to 1 (complete cytosine methylation).

## Estimation of epigenetic age

To generate the epigenetic ageing measures in H&N5000, we uploaded DNAm data for a subset of CpG sites from the Illumina EPIC array (n=27,523) to the online DNAm Age Calculator <https://dnamage.genetics.ucla.edu/> developed by Steve Horvath’s laboratory. This subset of sites was chosen based on the list of 30,085 CpGs listed in the “datMiniAnnotation3.csv” file available for “Advanced Analysis” on the DNA Methylation Age Calculator website. 2,562 CpG sites were missing due to probe discrepancy between the Illumina EPIC platform and Illumina 450K platform, the latter of which was used to derive some of the epigenetic clocks. We also uploaded an annotation file, containing data on chronological age, sex and tissue type for the samples. We were able to generate the following epigenetic ageing measures for 440 individuals (following the notation of previous publications): intrinsic epigenetic age acceleration based on Horvath’s multi-tissue predictor (*IEAA*) ^2^; intrinsic epigenetic age acceleration based on Hannum’s predictor (*IEAAHannum*) ^3^; extrinsic epigenetic age acceleration (*EEAA*), an enhanced version based on Hannum’s method, which up-weights the contribution of blood cell composition ^4^; PhenoAge (*AgeAccelPheno*) ^5^ and GrimAge (*AgeAccelGrim*) ^6^. *AgeAccelPheno* and *AgeAccelGrim* can be considered as measure of extrinsic ageing on the basis of estimators used ^6 7^. Intrinsic epigenetic age acceleration (IEAA) is independent of changes in blood cell composition while extrinsic epigenetic age acceleration (EEAA) incorporates age-related changes in blood cell composition and by this definition is able to capture aspects of immunosenescence ^4^.

*Generation of the DNAm mortality predictor (ZhangScore) in H&N5000*

The DNAm risk score for mortality was generated using the equation described in ^8^. Two of the CpGs included in the score were not present in the DNA methylation data, owing to probe discrepancy between the Illumina450k and EPIC arrays. These were cg06126421 and cg23665802. The mortality risk score was therefore calculated as:

*cg05575921*(-0.92224) + cg06126421*(-1.70129) + cg08362785*( 2.71749) + cg10321156*(-0.02073) + cg14975410*(-0.04156) + cg19572487*(-0.28069) + cg24704287*(-2.98637) + cg25983901*(-1.80325).*

*Multiple imputation*

Several of our covariates had some missing data, particularly BMI as this measure was not initially collected at recruitment. Excluding individuals with missing covariate data would have reduced statistical power to detect an association between the epigenetic age measures and survival, and so multiple imputation (MI) was performed. Previous work suggests that MI provides unbiased results in situations where data are missing at random (MAR) ^8^, i.e., any systematic differences between the observed and missing data can be explained by associations with the observed data. Missing values were imputed using the ICE package for multiple chained equations in Stata ^9^. Twenty imputed datasets were generated and analysed separately. The multiple sets of results were combined using ‘Rubin’s rules’ ^10^. The imputation models contained all the variables in the analysis model and the Nelson–Aalen estimator of the cumulative hazard. As a sensitivity analysis, we created a complete case dataset including only those participants with data available for our covariates of interest, and analysed as above ^11^.

*Flexible parametric survival models*

Survival models were fitted using the methods of Royston and Parmar ^12 13^, which model the baseline hazard (on the log-cumulative hazard scale) using restricted cubic splines ^14^. These are known as flexible parametric survival models. Unlike Cox models, these models permit absolute measures of effect to be estimated at all time points, rather than just at event times, ^15^ and thus it is easy to obtain predictions. The Royston and Parmar models were fitted using maximum likelihood estimation via the ‘stpm2’ command in Stata. The spline complexity for the baseline hazard which best fits the data was investigated visually and through model fit statistics (Akaike Information Criterion [AIC] and Bayesian Information Criterion [BIC]). We considered possible degrees of freedom (df) ranging from 1 to 5 df (for a model with no variables included). Using the hazard function plots and the AIC and BIC as a guide, 2 df were deemed sufficient. Non-linear relationships with continuous predictors were considered using the multivariable fractional polynomial (MFP) algorithm described by Sauerbrei and Royston ^16^ and implemented in Stata using the ‘mfp’ command.

Refertences

1. Min J, Hemani G, Davey Smith G, et al. Meffil: efficient normalisation and analysis of very large DNA methylation samples. *bioRxiv* 2017 doi: 10.1101/125963

2. Horvath S. DNA methylation age of human tissues and cell types. *Genome Biol* 2013;14(10):R115. doi: 10.1186/gb-2013-14-10-r115

3. Hannum G, Guinney J, Zhao L, et al. Genome-wide methylation profiles reveal quantitative views of human aging rates. *Mol Cell* 2013;49(2):359-67. doi: 10.1016/j.molcel.2012.10.016

4. Chen BH, Marioni RE, Colicino E, et al. DNA methylation-based measures of biological age: meta-analysis predicting time to death. *Aging (Albany NY)* 2016;8(9):1844-65. doi: 10.18632/aging.101020 [published Online First: 2016/10/01]

5. Levine ME, Lu AT, Quach A, et al. An epigenetic biomarker of aging for lifespan and healthspan. *Aging (Albany NY)* 2018;10(4):573-91. doi: 10.18632/aging.101414 [published Online First: 2018/04/21]

6. Lu AT, Quach A, Wilson JG, et al. DNA methylation GrimAge strongly predicts lifespan and healthspan. *Aging (Albany NY)* 2019;11(2):303-27. doi: 10.18632/aging.101684 [published Online First: 2019/01/23]

7. Horvath S, Raj K. DNA methylation-based biomarkers and the epigenetic clock theory of ageing. *Nat Rev Genet* 2018;19(6):371-84. doi: 10.1038/s41576-018-0004-3 [published Online First: 2018/04/13]

8. Hughes RA, Heron J, Sterne JAC, et al. Accounting for missing data in statistical analyses: multiple imputation is not always the answer. *International journal of epidemiology* 2019 doi: 10.1093/ije/dyz032 [published Online First: 2019/03/18]

9. Royston P. Multiple imputation of missing values: further update of ice, with an emphasis on categorical variables. *Stata Journal* 2009;9:466–77.

10. Rubin D, Schenker N Multiple Imputation for Interval Estimation from Simple Random Samples with Ignorable Nonresponse. *Journal of the American Statistical Association* 1989;81:366–74.

11. White IR, Royston P. Imputing missing covariate values for the Cox model. *Stat Med* 2009;28(15):1982-98. doi: 10.1002/sim.3618

12. Royston P. Flexible parametric alternatives to the Cox model, and more. *Stata Journal* 2001;1:1-28.

13. Royston P, Parmar MK. Flexible parametric proportional-hazards and proportional-odds models for censored survival data, with application to prognostic modelling and estimation of treatment effects. *Stat Med* 2002;21(15):2175-97. doi: 10.1002/sim.1203 [published Online First: 2002/09/05]

14. Ensor J, Riley RD, Jowett S, et al. Prediction of risk of recurrence of venous thromboembolism following treatment for a first unprovoked venous thromboembolism: systematic review, prognostic model and clinical decision rule, and economic evaluation. *Health Technol Assess* 2016;20(12):i-xxxiii, 1-190. doi: 10.3310/hta20120 [published Online First: 2016/02/18]

15. Ng R, Kornas K, Sutradhar R, et al. The current application of the Royston-Parmar model for prognostic modeling in health research: a scoping review. *Diagn Progn Res* 2018;2:4. doi: 10.1186/s41512-018-0026-5 [published Online First: 2018/02/07]

16. Royston P SW. Multivariable Model-Building: A Pragmatic Approach to Regression Analysis Based on Fractional Polynomials for Modelling Continuous Variables. Chichester: John Wiley & Sons, Ltd 2008.
